# Temperature-dependent sex expression in cucurbits and beyond: mechanisms, reproductive plasticity, and breeding implications

**DOI:** 10.3389/fpls.2026.1902742

**Published:** 2026-08-26

**Authors:** Yang Tao, Cheng Chang, Yujie Yang, Xiao Huang, Min Sun, Ziwei Zhu, Yixi Yang, Rui Li, Bingliang Liu, Qi Zhao

**Affiliations:** 1 Engineering Research Center of Sichuan-Xizang Traditional Medicinal Plant, College of Food and Biological Engineering, Chengdu University, Chengdu, China; 2 Sichuan-Xizang Medicinal Resource Breeding and Standardization Team, Institute for Advanced Study, Chengdu University, Chengdu, China; 3 Natural Products Chem-Bio Innovation Center, Chengdu University, Chengdu, China; 4 Institute of Urban Agriculture, Chinese Academy of Agricultural Sciences, National Agricultural Science & Technology Center, Chengdu, China

**Keywords:** cucurbits, heat stress, reproductive plasticity, sex-regulatory networks, temperature-dependent sex expression

## Abstract

Global warming and increasingly frequent heat extremes threaten plant reproductive success and yield stability. Beyond causing general reproductive heat injury, such as pollen sterility, stigma dysfunction, and fertilization failure, temperature can also modify floral sex expression during early developmental windows. Temperature-dependent sex expression encompasses temperature-responsive changes in floral sex fate, floral sex ratios, and sex-expression patterns, and represents an underexplored dimension of reproductive plasticity under thermal stress. Here, we clarify the distinctions among sex determination, sex differentiation, sex expression, and sexual plasticity, and distinguish temperature-dependent sex expression from general reproductive heat injury. We focus on cucumber and melon as the most informative model systems and synthesize evidence for temperature-sensitive phenotypes, ethylene-centered ACS/ACO–WIP–ERF networks, GA-associated pathways, and epigenetic regulation involving DNA methylation and small RNAs. Importantly, we emphasize that direct causal links between defined temperature cues and individual sex-regulatory components remain incompletely resolved. Evidence from dioecious and non-model plants, including spinach and *Cannabis*, is critically evaluated to distinguish direct temperature-induced changes in floral sex fate from hormone-responsive sexual plasticity and sex-specific stress responses. We further assess the context-dependent adaptive potential and constraints of sexual plasticity and identify priorities involving causal validation, single-cell and spatial omics, natural variation, and field-relevant temperature regimes. This review provides a critical framework for understanding temperature-responsive sex expression and for translating mechanistic advances into temperature-stable breeding, hybrid seed production, and controlled-environment cultivation.

## 1 Introduction

Global warming and the increasing frequency of extreme heat events are reshaping the environmental context in which plants grow, develop, and reproduce, and are likely to become long-term challenges for agroecosystems (Lesk et al., 2022). Plant scientists have also identified how plants maintain growth, reproduction, and adaptive capacity under climate change as an important question for future research (Armstrong et al., 2023; Shimizu et al., 2011). In this context, the responses of plant reproductive development to temperature changes deserve particular attention, because reproductive stages are often more vulnerable to heat stress than vegetative growth (Zinn et al., 2010). However, the effects of temperature on plant reproduction are not limited to conventional reproductive heat injury, such as pollen sterility, stigma dysfunction, or fertilization failure. In many plants with unisexual flowers or flexible sex expression, temperature may also act as a developmental cue during critical windows of floral primordium differentiation or sex determination, thereby altering the ratio of male to female flowers, floral organ fate, or even sex expression at the level of individual plants or branches (Xie et al., 2023). Temperature therefore does not merely damage already differentiated reproductive organs; it can also reshape sex-expression programs at earlier developmental stages.

Plant sexual systems are highly diverse, ranging from hermaphroditism and monoecy to dioecy, and plants generally exhibit considerable developmental and environmental plasticity in sex expression (Pannell, 2017). Sex determination and sex expression are therefore not governed solely by genetic factors, but can also be modulated by temperature, photoperiod, nutrient availability, and endogenous hormonal signals (Lai et al., 2018). Such plasticity has direct agricultural relevance because changes in floral sex ratios can alter female flower production, fruit set, early yield, and production stability, particularly in cucurbit and other horticultural crops grown under warm-season or protected-cultivation conditions (Li et al., 2021; Luo et al., 2023; Petry et al., 2016; Wien et al., 2004). Understanding the developmental and molecular basis of temperature-sensitive sex expression may therefore contribute to breeding cultivars with more stable reproductive performance and to improving environmental management in controlled production systems.

The Cucurbitaceae provides a particularly informative model for studying environmentally responsive sex expression because it combines diverse sexual systems with pronounced developmental and hormonal plasticity. Monoecy, in which separate male and female flowers are produced on the same plant, is the most common sexual system in cucurbits and occurs widely in cucumber, watermelon, pumpkin, and squash. However, andromonoecious, gynoecious, androecious, hermaphroditic, and other intermediate forms also occur, with andromonoecy being particularly common in cultivated melon (Dong et al., 2025; Li et al., 2019). In cucumber and melon, floral meristems initially form both stamen and carpel primordia, after which unisexual flowers arise through the selective arrest of one reproductive organ type (Bai et al., 2004; Boualem et al., 2015; Saito et al., 2007). This bipotential developmental stage creates a responsive window during which environmental and hormonal signals can modify floral sex fate (Lai et al., 2018; Yamasaki et al., 2003).

Ethylene provides the best-characterized molecular framework for sex determination in cucumber (Li et al., 2019; Zhang et al., 2021). The major Female, Monoecious, and Androecious loci correspond to the ACC synthase genes *CsACS1G*, *CsACS2*, and *CsACS11*, respectively (Boualem et al., 2015; Li et al., 2009; Zhang et al., 2021). *CsACS11* and the ACC oxidase gene *CsACO2* contribute to early ethylene production required for carpel initiation and female flower development, whereas the C2H2 zinc-finger transcription factor *CsWIP1* acts as a masculinizing regulator within this network (Chen et al., 2016; Hu et al., 2017). Subsequent *CsACS2* activity promotes stamen arrest and the development of functionally female flowers, whereas early expression of *CsACS1G* establishes a dominant female developmental pathway and gynoecy (Saito et al., 2007; Zhang et al., 2021). Together, these genes form an ethylene-centered regulatory network that provides a mechanistic basis for investigating how environmental signals influence floral sex fate. However, most functional evidence for this network has been obtained through genetic, gene-editing, or hormonal perturbations under standard growth conditions, and the direct connections between defined temperature regimes and individual sex-regulatory nodes remain incompletely resolved.

Despite recent progress, three major gaps remain. First, mechanistic evidence is uneven across plant systems: functional studies are concentrated in cucurbits, whereas most non-cucurbit research documents hormone-responsive plasticity or sex-specific stress responses. Second, the pathways connecting temperature perception with hormonal, epigenetic, and sex-regulatory networks remain poorly resolved. Third, simplified constant-temperature experiments limit translation to field-relevant conditions, including heatwaves, nocturnal warming, fluctuating day–night temperatures, and compound stresses (Jagadish et al., 2021; Lesk et al., 2022).

In this review, we examine temperature-dependent sex expression as an underexplored dimension of reproductive plasticity under heat stress. We first clarify the distinctions among sex determination, sex differentiation, sex expression, and sexual plasticity, and distinguish temperature-dependent sex expression from general reproductive heat injury. We then use cucurbit crops as the central mechanistic framework, while critically evaluating complementary evidence from dioecious and non-model systems, including spinach and *Cannabis*. By integrating developmental, hormonal, genetic, epigenetic, ecological, and breeding perspectives, we assess the strength and limitations of current evidence and outline how mechanistic advances could support temperature-stable breeding, hybrid seed production, and controlled-environment cultivation.

## 2 Conceptual framework: distinguishing sex expression from reproductive heat injury

### 2.1 Sex determination, sex differentiation, sex expression, and sexual plasticity

Sex determination, sex differentiation, sex expression, and sexual plasticity refer to distinct but developmentally interconnected levels of plant sexual development. Distinguishing these concepts is essential for interpreting how temperature influences reproductive phenotypes.

Sex determination refers to the molecular and developmental processes that establish male, female, or bisexual identity at the level of an individual plant or floral organ. Plant sex-determination systems are highly diverse, ranging from genetically controlled mechanisms involving sex chromosomes or sex-determining regions to developmental pathways that can be strongly modulated by environmental factors such as temperature, photoperiod, and nutrient availability (Li et al., 2025; Pannell, 2017). Studies of dioecious plants, including asparagus and kiwifruit, further indicate that different lineages have repeatedly recruited distinct genes and regulatory modules during the evolution of sexual systems (Leite Montalvão et al., 2020).

Sex differentiation is the developmental execution of sex determination, during which reproductive primordia follow a specified sexual trajectory through the continued development or selective arrest of stamens and carpels under transcriptional and hormonal control. This process involves spatially and temporally regulated cell-fate specification and the formation or selective arrest of sex-specific reproductive organs (You et al., 2025).

Sex expression is the observable manifestation of these developmental processes. It encompasses floral sex identity, the number and spatial distribution of male, female, or bisexual flowers, and the stability of these phenotypes during plant development. Sex expression directly influences reproductive efficiency and agronomic performance (Pan et al., 2018; Pannell, 2017).

Sexual plasticity refers to the capacity of the same genotype to produce different sexual phenotypes under different environmental conditions. Temperature, photoperiod, and nutrient availability may influence sex expression by interacting with genetically programmed developmental pathways through hormonal or epigenetic regulation (Li et al., 2025). Such plasticity can increase environmental responsiveness, but it is adaptive only when it improves reproductive performance or fitness under the inducing conditions.

### 2.2 Temperature-dependent sex expression versus general reproductive heat injury

**Figure 1:**
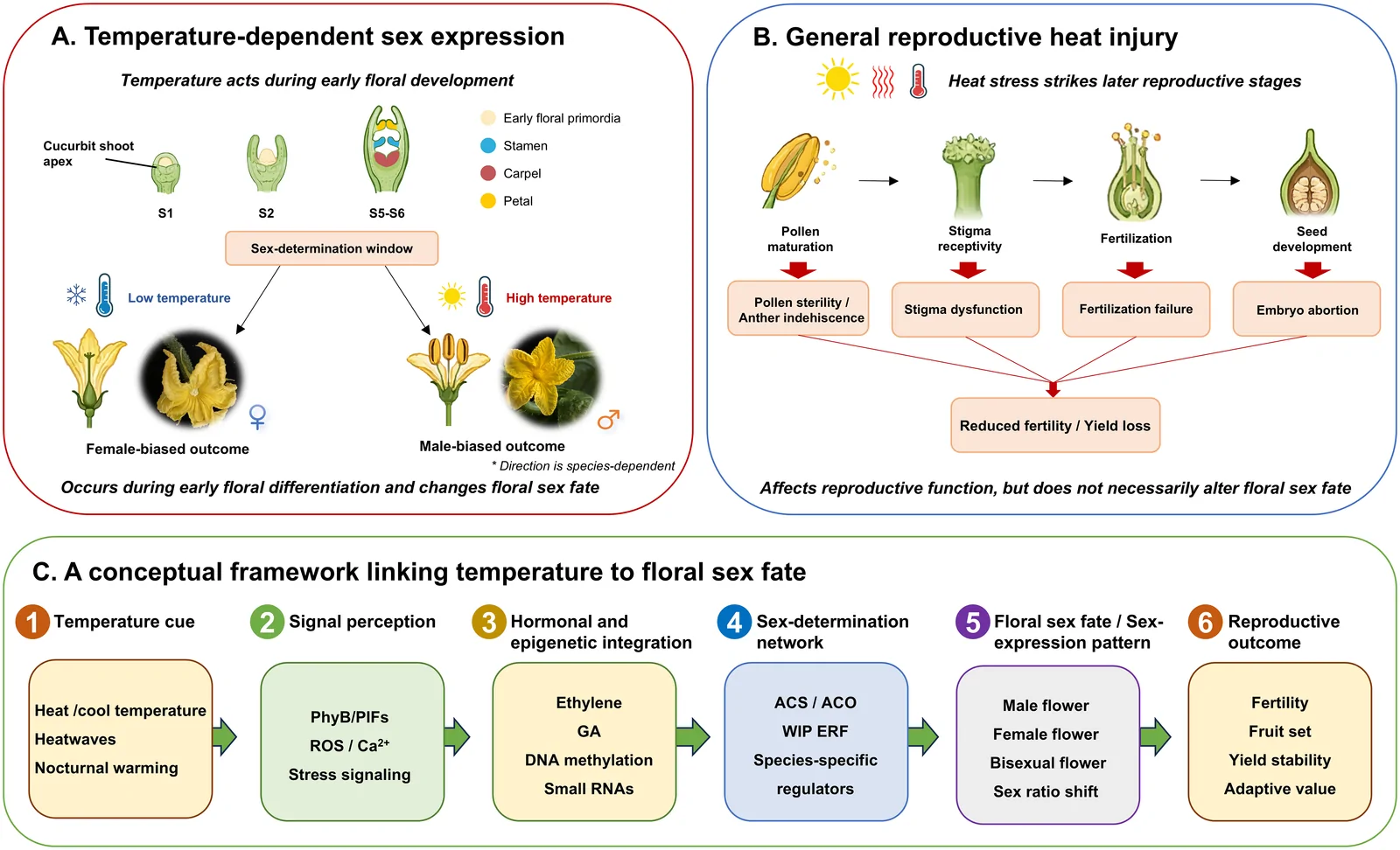
Conceptual framework distinguishing temperature-dependent sex expression from general reproductive heat injury in plants. **(A)** Schematic representation of temperature-dependent sex expression, using cucumber as an illustrative model. During early floral development, cucumber floral buds initially form both stamen and carpel primordia, after which temperature cues acting within responsive developmental windows may influence the selective development or arrest of reproductive organs. These effects can alter floral sex fate or the relative production of male, female, and bisexual flowers. The direction and magnitude of the response depend on species, genotype, developmental stage, and temperature regime. The developmental staging of cucumber floral organs was based on Dong et al. (2025). **(B)** Schematic representation of general reproductive heat injury. Heat stress acting at later reproductive stages mainly impairs reproductive organ function, including pollen viability, stigma receptivity, fertilization, and embryo or seed development, but does not necessarily alter floral sex fate. **(C)** A conceptual framework linking temperature cues to floral sex fate. Temperature signals are perceived and integrated through signaling, hormonal, and epigenetic pathways, which may converge on sex-determination or floral organ development networks to regulate floral sex expression and ultimately shape reproductive outcomes. Some graphical elements were hand-drawn by the authors; others were created with the assistance of BioGDP.com (Jiang et al., 2025) and are used with permission.

Although temperature-dependent sex expression and general reproductive heat injury both involve temperature effects on reproduction, they differ in developmental timing, mode of action, and biological outcome (Lai et al., 2018). Temperature-dependent sex expression occurs when floral sex fate remains developmentally plastic, particularly during floral primordium initiation or sex differentiation. During these windows, temperature may influence male or female development through hormonal balance, epigenetic states, and sex-determination networks (Lai et al., 2017; Li et al., 2019). The resulting phenotypes may include changes in floral sex identity, male-to-female flower ratios, bisexual flower formation, or sex-expression stability (**Figure 1A**).

However, an observed shift in the male-to-female flower ratio should not automatically be interpreted as a change in floral sex fate. Similar plant- or population-level patterns may result from altered meristem identity, selective abortion of one flower type, differential flowering intensity, or sex-specific heat sensitivity. A change in the observed sex ratio is therefore a phenotypic outcome rather than direct evidence of a particular developmental mechanism. Distinguishing among these processes is essential for mechanistic interpretation.

By contrast, general reproductive heat injury usually occurs after floral sex identity has already been established, including during meiosis, pollen maturation, stigma receptivity, fertilization, and embryo development (**Figure 1B**) (Resentini et al., 2023; Zinn et al., 2010). High temperature may cause pollen sterility, stigma dysfunction, fertilization failure, or abnormal embryo development through membrane injury, reactive oxygen species accumulation, disruption of protein homeostasis, and hormonal imbalance (Rieu et al., 2017). These responses reduce the viability or function of reproductive organs without necessarily redefining their sexual identity.

Because temperature sensitivity varies among species, genotypes, developmental stages, day/night regimes, and treatment durations, no universal threshold defines temperature-dependent sex expression. In this review, “high” and “low” temperatures refer to conditions above or below the developmental optimum or experimental control used in individual studies; relatively cool conditions that favor female development should not be conflated with chilling stress. A temperature response should therefore be classified as temperature-dependent sex expression only when evidence indicates that temperature alters floral sex identity or sex-expression patterns during a developmental window in which sexual fate remains plastic.

### 2.3 A framework linking temperature signals to floral sex fate

Temperature-dependent sex expression can be conceptualized as a multilevel process connecting temperature perception, hormonal and epigenetic regulation, sex-determination networks, and floral sex fate (**Figure 1C**) (Ding et al., 2020; Pawełkowicz et al., 2019). Temperature-responsive pathways involving calcium signals, reactive oxygen species, heat-shock proteins, and transcriptional regulators may influence hormone biosynthesis and signaling, particularly ethylene- and GA-associated pathways (Kerbler and Wigge, 2023; Qi et al., 2022). Temperature may also be associated with changes in DNA methylation, chromatin states, and small RNA-mediated regulation, which could modify the activity of sex-related genes (Lämke and Bäurle, 2017). These regulatory layers may ultimately converge on floral organ-development and sex-determination networks to influence the developmental trajectories of reproductive primordia (Zhang et al., 2017). This framework should be regarded as an integrative working model because its upstream temperature-to-sex links, epigenetic causality, and applicability beyond cucurbits remain incompletely validated.

## 3 Mechanistic insights from cucurbits and comparative plant systems

### 3.1 Temperature-responsive sex expression and developmental windows in cucurbits

**Figure 2:**
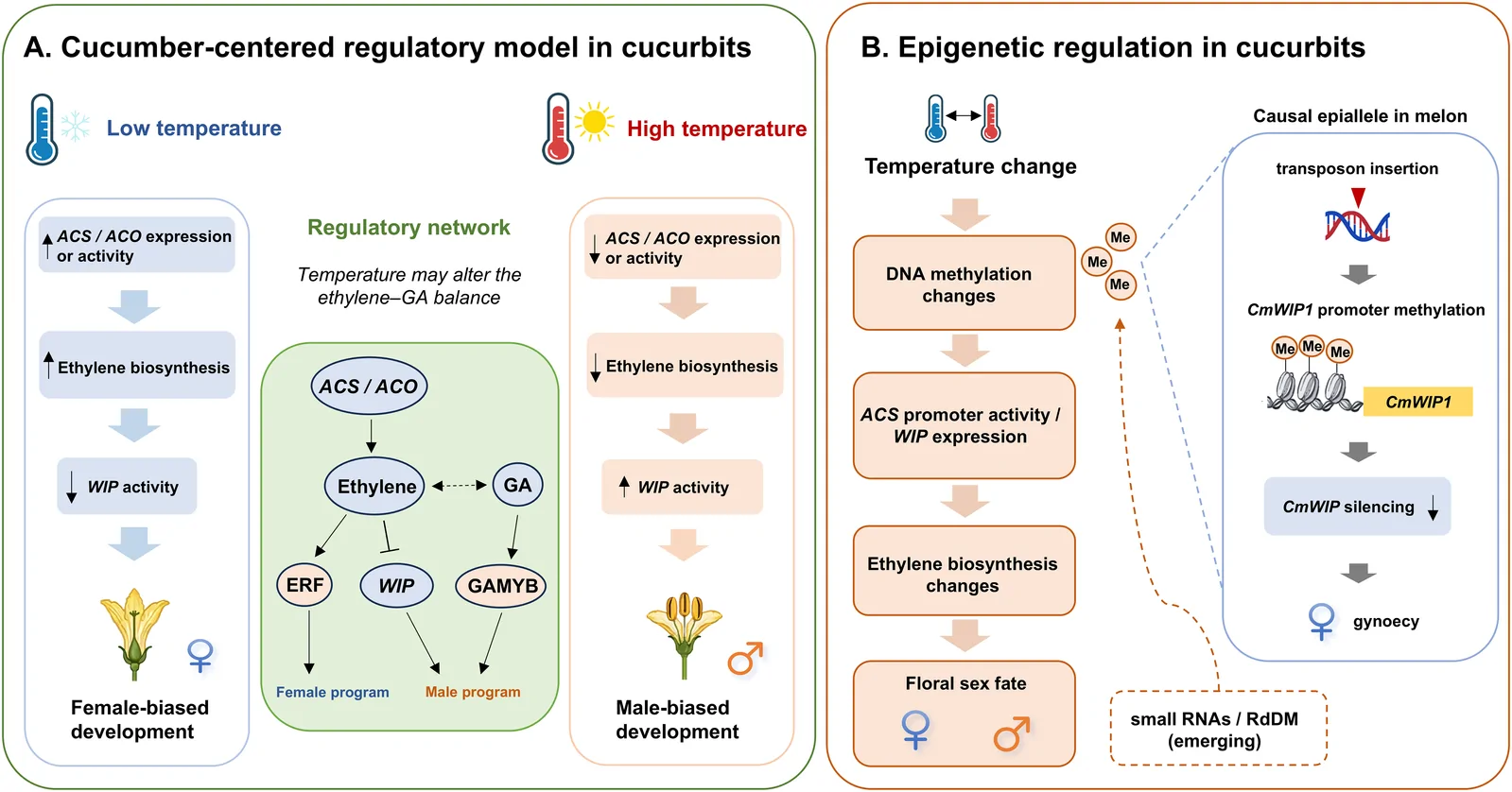
Hormonal, genetic, and epigenetic regulation of temperature-dependent sex expression in cucurbits. **(A)** Cucumber-centered hormonal and genetic framework. Temperature may influence floral sex expression by altering ethylene biosynthesis and its interaction with gibberellin-associated pathways. Lower temperature is generally associated with female-biased development and may enhance ethylene-associated feminizing signals, whereas higher temperature tends to favor male-biased sex expression. ACS/ACO, WIP, ERF, and GAMYB represent key regulatory modules involved in ethylene biosynthesis, masculinizing activity, ethylene-responsive transcription, and GA-associated regulation, respectively. **(B)** Epigenetic regulation and current evidence gaps. In cucumber, temperature-associated changes in DNA methylation, particularly CHH methylation and small RNA-related RNA-directed DNA methylation, correlate with altered expression of sex- and ethylene-related genes and shifts in floral sex expression. These relationships remain largely associative. By contrast, transposon-associated promoter methylation and stable silencing of *CmWIP1* provide causal evidence that an epigenetic state can determine floral sex in melon, although this mechanism has not been shown to be dynamically induced by temperature. Solid lines indicate regulatory relationships supported by genetic, physiological, or functional evidence, whereas dashed lines indicate indirect, emerging, or putative links that require further causal validation. Some graphical elements were hand-drawn by the authors.

Cucumber and melon provide the most extensive phenotypic evidence for environmentally responsive floral sex expression because their sexual phenotypes are genetically tractable and sensitive to temperature, photoperiod, and hormonal treatments (Li et al., 2019; Pawełkowicz et al., 2019). In cucumber, relatively cool temperatures and short photoperiods generally increase the proportion of nodes bearing female flowers, whereas warmer temperatures and long photoperiods favor male-biased sex expression (**Figure 2A**). A five-year survey of 359 cucumber accessions showed that seasonal variation in femaleness is widespread, although the magnitude and direction of the response vary among genotypes (Lai et al., 2018). Hormonal treatments further indicate that this environmental responsiveness is restricted to an early developmental window. Application of the ethylene-releasing compound ethephon or the ethylene-biosynthesis inhibitor aminoethoxyvinylglycine altered sex differentiation most effectively around the stage of stamen primordium development, but had substantially weaker effects after floral organ identity had become morphologically fixed (Yamasaki et al., 2003). Although these experiments do not directly identify a temperature-sensing mechanism, they define a hormone-responsive developmental period during which temperature-dependent changes in signaling could alter floral sex fate.

Comparable temperature responses have been reported in other cucurbits, although the evidence is less mechanistically integrated. In watermelon, a daytime temperature of 32 °C inhibited female flower development relative to 27 °C, whereas elevated greenhouse temperatures were associated with reduced ethylene production and increased bisexual flower formation (Manzano et al., 2014; Rudich and Peles, 1976). In *Cucurbita* species, high temperature can delay female flowering and increase female flower-bud abortion or failure to reach anthesis (Wien et al., 2004). These latter responses require cautious interpretation because selective abortion after floral sex identity has been established may represent reproductive heat injury rather than a temperature-induced change in floral sex fate.

Overall, temperature responses in cucurbits vary with genotype, developmental timing, and the phenotype measured; observed sex-ratio shifts should therefore be resolved into altered sex determination, flower initiation, or selective floral abortion.

### 3.2 Ethylene as a central regulator of sex determination in cucurbits

Building on the ethylene-centered framework introduced above, functional studies in cucurbits have resolved key genetic controls of carpel initiation and stamen arrest (**Figure 2A**). The major Female, Monoecious, and Androecious loci correspond to the ACC synthase genes *CsACS1G*, *CsACS2*, and *CsACS11*, respectively. The ACC oxidase gene *CsACO2*, which catalyzes the final step of ethylene biosynthesis, is also essential for female flower development. Loss of *CsACO2* produces an androecious phenotype, demonstrating that local ethylene production is required for carpel initiation and development (Chen et al., 2016). Conversely, CRISPR/Cas9 disruption of the masculinizing transcription factor *CsWIP1* produces predominantly gynoecious plants, showing that *CsWIP1* normally suppresses the female developmental pathway (Hu et al., 2017).

In monoecious cucumber, *CsACS11* and *CsACO2* exhibit overlapping expression in the carpel region of floral buds destined to become female. Their coordinated activity is proposed to generate an early ethylene signal that represses *CsWIP1* and permits activation of *CsACS2*. Subsequent *CsACS2* activity promotes stamen arrest and the formation of a functionally female flower. In gynoecious cucumber, promoter rearrangement enables earlier expression of *CsACS1G*. CRISPR/Cas9, mutagenesis, and transformation analyses demonstrated that *CsACS1G*, rather than neighboring genes within the F-locus duplication, is responsible for gynoecy. Its early spatial overlap with *CsACO2* enables ethylene production before *CsACS11* would normally become active, thereby establishing a dominant female developmental pathway (Chen et al., 2016; Zhang et al., 2021).

Ethylene signaling may further reinforce this biosynthetic network through transcriptional feedback. *CsERF110* and its melon ortholog *CmERF110* are induced by ethylene. Yeast one-hybrid and ChIP-PCR assays showed that CsERF110 binds the *CsACS11* promoter, whereas transient-expression assays demonstrated that CsERF110 and CmERF110 enhance the activities of the *CsACS11* and *CmACS11* promoters, respectively (Tao et al., 2018). These results support an ethylene–ERF110–ACS11 positive-feedback module, although its necessity for temperature-responsive sex expression remains to be established using stable loss-of-function materials.

Melon shares an ethylene-centered framework but has a distinct genetic architecture. *CmWIP1* promotes male flower development by inducing carpel abortion and repressing the female developmental pathway. Stable silencing of *CmWIP1* releases carpel development and produces gynoecy, providing direct evidence that this masculinizing factor controls floral sex fate (Martin et al., 2009). The androecy regulator *CmACS11* functions upstream of this pathway, whereas ethylene produced in carpel primordia induces *CmHB40* expression in developing stamens. CmHB40 subsequently represses genes required for stamen development, thereby promoting female flower formation (Boualem et al., 2015; Rashid et al., 2023). These findings reveal both genetic and inter-organ ethylene signaling during melon sex determination.

Ethylene also contributes to female flower development in watermelon and *Cucurbita*. In watermelon, ethylene is required for stamen arrest and normal female flower development, although its effects on the transition between male and female flowering appear to depend on developmental and environmental context (Manzano et al., 2014). In *Cucurbita pepo*, mutations affecting the ethylene receptors *CpETR1A* and *CpETR2B* or the ethylene-biosynthesis gene *CpACO1A* disrupt female flower initiation or development (Cebrián et al., 2022; Garcia et al., 2020).

### 3.3 GA-associated regulation and its relationship with ethylene

GA generally shifts cucumber sex expression toward maleness, but its relationship with ethylene cannot be reduced to simple hormonal antagonism. Available evidence instead suggests the presence of both ethylene-independent and potentially ethylene-associated regulatory branches. *CsGAMYB1* is induced by GA and is preferentially expressed in male floral organs. RNAi-mediated reduction of *CsGAMYB1* decreased the proportion of male-flower nodes from 75.7% to 45% and increased female-flower nodes from 24.3% to 55%. Because ethylene production and the expression of *CsACS1G* and *CsACS2* were not significantly altered in the RNAi lines, these findings support an ethylene-independent GA–CsGAMYB1 pathway promoting male flower development (Zhang et al., 2014). By contrast, transcriptomic analyses following GA treatment revealed altered expression of *CsACS2*, *CsETR1*, and several ERF genes, suggesting that GA may also influence sex expression through an ethylene-associated branch (Zhang et al., 2017). However, this second branch remains based largely on expression responses and requires direct genetic validation.

Current evidence therefore supports a functionally validated GA–CsGAMYB1 branch together with a less well-resolved interaction between GA and ethylene signaling. Whether temperature acts through either of these GA-associated pathways, and whether GA contributes directly to temperature-induced changes in floral sex fate, remain important unresolved questions.

### 3.4 Epigenetic and small-RNA regulation: associations and causal gaps

Beyond hormonal regulation, epigenetic and small-RNA pathways represent plausible interfaces between environmental temperature and cucurbit sex-regulatory networks, but causal epialleles must be distinguished from temperature-associated molecular changes whose functional roles remain unverified (**Figure 2B**).

In cucumber, low-temperature treatment induces extensive changes in genome-wide DNA methylation, particularly at CHH sites and in transposable-element-rich regions near genes. Changes in 24-nt sRNA abundance correlate positively with CHH methylation changes, and several putative RdDM components are transcriptionally reduced under low temperature, supporting the involvement of an sRNA-guided RdDM pathway in temperature-responsive methylome remodeling (Lai et al., 2017). A subsequent study showed that high temperature and long photoperiod both alter the cucumber methylome, but affect partly distinct genomic regions, suggesting that these environmental cues may converge on epigenetic regulation through different targets (Lai et al., 2018). Nevertheless, neither study demonstrated that individual differentially methylated regions are necessary or sufficient for the observed changes in floral sex expression.

The melon *CmWIP1* locus provides stronger causal evidence for epigenetic control of floral sex. Transposon-associated methylation of the *CmWIP1* promoter stably silences this masculinizing gene and shifts monoecious or andromonoecious developmental programs toward gynoecy (Martin et al., 2009). However, this heritable epiallele is mechanistically distinct from dynamic temperature-induced methylation: it demonstrates that epigenetic silencing can determine floral sex, but not that temperature regulates *CmWIP1* through the same process.

Evidence for miRNA-mediated regulation is more exploratory. Small-RNA, degradome, and transcriptome analyses of cucumber shoot apices identified eight known and 16 novel miRNAs responsive to temperature and photoperiod. Several degradome-supported targets were associated with hormone signaling and developmental regulation, including candidate miRNA–target modules involving miR156/157–SBP factors and a cucumber-specific miRNA targeting an ethylene-responsive transcription factor (Zhang et al., 2018). However, these findings are based largely on differential abundance, target prediction, and degradome-supported cleavage; no individual miRNA or 24-nt sRNA has yet been shown by genetic perturbation or complementation to be required for temperature-induced changes in floral sex fate.

Thus, epigenetic and small-RNA pathways remain promising but incompletely validated components of temperature-dependent sex expression. Causal testing will require targeted methylation or demethylation, disruption of RdDM components, editing of miRNA target sites, and phenotyping under defined temperature-sensitive developmental windows.

### 3.5 Evidence beyond cucurbits: complementary systems and current limitations

Beyond cucurbits, evidence is more heterogeneous and addresses several related, but mechanistically distinct, forms of sexual plasticity. In spinach, exogenous GA can masculinize genetically female plants. Functional analyses identified the *SpGAI–SpSTM–SpPI* regulatory module as a mediator of male organ development, providing direct evidence for GA-responsive sexual plasticity (West and Golenberg, 2018; Zhang et al., 2024). Sex-associated DNA methylation differences and spatially resolved transcriptional programs have also been identified during spinach sex differentiation (Jia et al., 2024; You et al., 2025). However, whether temperature alters these regulatory processes or induces comparable sex conversion remains unknown. Spinach therefore provides a mechanistically relevant model of hormone-induced sexual plasticity, but not yet an established model of temperature-dependent sex expression.

Similarly, *Cannabis sativa* has an XX/XY genetic sex-determination system, but manipulation of ethylene biosynthesis or signaling can induce floral phenotypic sex reversal in both genetic sexes. Orthology-based analyses have reconstructed the principal ethylene biosynthesis and signaling components in *Cannabis*, while subsequent multi-omic analyses linked induced sex reversal to sex-specific ethylene-responsive transcriptional networks and genetic background (Monthony et al., 2024, 2026). These findings demonstrate that floral sexual identity remains hormonally plastic within a chromosomal sex-determination system, although direct evidence that temperature initiates this response is currently lacking.

Evidence from poplar, willow, and other woody dioecious plants is more indirect. Male and female individuals can differ in growth, photosynthesis, antioxidant protection, and water relations under drought and nocturnal warming (Liao et al., 2019; Peng et al., 2012). These studies reveal sex-specific environmental responses, but they concern differential stress tolerance rather than temperature-induced changes in floral sex fate.

As summarized in **Table 1**, cucumber currently provides the most complete integration of controlled-temperature phenotypes and functionally characterized sex-regulatory pathways. Spinach, *Cannabis*, and woody dioecious plants broaden the comparative context by revealing hormone-responsive plasticity or sex-specific environmental responses, but these systems do not yet provide mechanistically equivalent evidence for direct temperature-induced changes in floral sex fate.

**Table 1:** Comparative evidence for temperature-dependent sex expression and related sexual plasticity across plant systems.

Plant system	Relevant evidence	Mechanistic support	Key limitation	Evidence level	Representative references
Cucumber (*Cucumis sativus*)	Controlled temperature treatments alter male-to-female flower ratios; associated methylation and small-RNA changes have been reported	Ethylene-related sex regulators, including *ACS*, *ACO*, and *WIP*, have functional support	The complete pathway from temperature perception to floral sex fate remains unresolved	I	(Lai et al., 2018, 2017; Zhang et al., 2021)
Melon (*Cucumis melo*)	Sex expression is environmentally responsive	*CmACS7*, *CmACS11*, *CmWIP1*, and *CmHB40* regulate floral sex; the *CmWIP1* epiallele is functionally established	Direct temperature-dependent regulation of these components is insufficiently demonstrated	II	(Boualem et al., 2008, 2015; Martin et al., 2009; Rashid et al., 2023)
Watermelon (*Citrullus lanatus*)	Temperature affects female flower formation and sex ratios	Ethylene is involved in floral sex expression and female flower development	The causal link between temperature, ethylene, and sex fate is unclear	II	(Manzano et al., 2014; Rudich and Peles, 1976)
Pumpkin and squash (*Cucurbita* spp.)	High temperature delays female flowering and may increase female bud abortion	Ethylene biosynthesis and perception are required for female flower development	Temperature effects and ethylene mechanisms have not been causally integrated	II	(Cebrián et al., 2022; Garcia et al., 2020; Wien et al., 2004)
Spinach (*Spinacia oleracea*)	GA treatment can masculinize female plants	The *SpGAI–SpSTM–SpPI* module and sex-associated methylation patterns have been identified	Direct evidence for temperature-induced changes in floral sex fate is lacking	II	(Jia et al., 2024; You et al., 2025; Zhang et al., 2024)
*Cannabis sativa*	Manipulation of ethylene biosynthesis or signalling can induce floral sex reversal	Ethylene-responsive transcriptional networks are associated with sexual plasticity	The demonstrated trigger is hormonal rather than temperature based	II	(Monthony et al., 2024, 2026)
Poplar, willow, and other woody dioecious plants	Male and female plants show different responses to heat, drought, and warming	Sex-biased physiological and transcriptional responses have been reported	These studies concern sex-specific stress responses, not changes in floral sex identity	III	(Melnikova et al., 2017)

Evidence levels: Level I, controlled temperature treatments directly alter floral sex expression, and relevant sex-regulatory pathways have functional support; Level II, direct temperature-responsive phenotypes or functionally validated sexual-plasticity mechanisms are available, but the temperature-to-sex-fate connection remains incomplete; Level III, evidence is limited to sex-specific stress responses or molecular associations without demonstrated changes in floral sex identity.

## 4 Sexual plasticity: adaptive potential and constraints

Temperature-responsive sexual plasticity should not automatically be interpreted as adaptive. Here, we consider a response potentially adaptive only when it improves reproductive performance or fitness under the environmental conditions that induce it. In the absence of such evidence, it is more appropriately described as temperature-responsive plasticity or stress-induced developmental perturbation.

### 4.1 Environmental modulation of reproductive allocation

Temperature can alter allocation to pollen, ovules, fruits, and seeds by affecting carbon balance, respiration, hormonal status, and floral development. The reproductive consequences vary among sexual systems, genotypes, developmental stages, and resource environments (Scheepens et al., 2018). In cucurbits, temperature can alter reproductive allocation within individuals by shifting floral sex ratios. In sexually dimorphic species, environmental effects on population sex ratios may instead result from sex-specific survival, flowering intensity, or reproductive costs without changing floral identity (Varga and Soulsbury, 2020). Individual-level changes in floral sex fate are directly relevant to temperature-dependent sex expression, whereas population-level shifts provide complementary ecological evidence of sex-specific environmental responses.

The adaptive consequences of altered sex allocation are context dependent. Under high temperature or resource limitation, reduced investment in female function may lower reproductive costs in some ecological settings. In crop systems, however, the same shift may reflect developmental disruption and reduce female flower production, fruit set, and yield (Sánchez Vilas and Pannell, 2014).

Temperature rarely acts in isolation. In cucumber, temperature and photoperiod both influence floral sex expression, but transcriptomic and methylomic analyses suggest that they act through partly distinct regulatory and epigenetic routes (Lai et al., 2018). Other environmental constraints may independently reshape reproductive allocation or sexual phenotypes. For example, drought has sex-specific effects on flower production and reproductive resources in *Cucurbita pepo*, with female flowers generally showing greater sensitivity, whereas nutrient availability can regulate resource allocation and year-to-year sex expression in the gender-diphasic plant *Lilium concolor* var. *megalanthum* (Barman et al., 2024; Chen et al., 2023). However, direct multifactorial studies testing how temperature interacts with photoperiod, water availability, or nutrient status to determine floral sex fate remain limited, making such experiments an important priority for future research.

### 4.2 Evolutionary trade-offs between stability and plasticity

**Figure 3:**
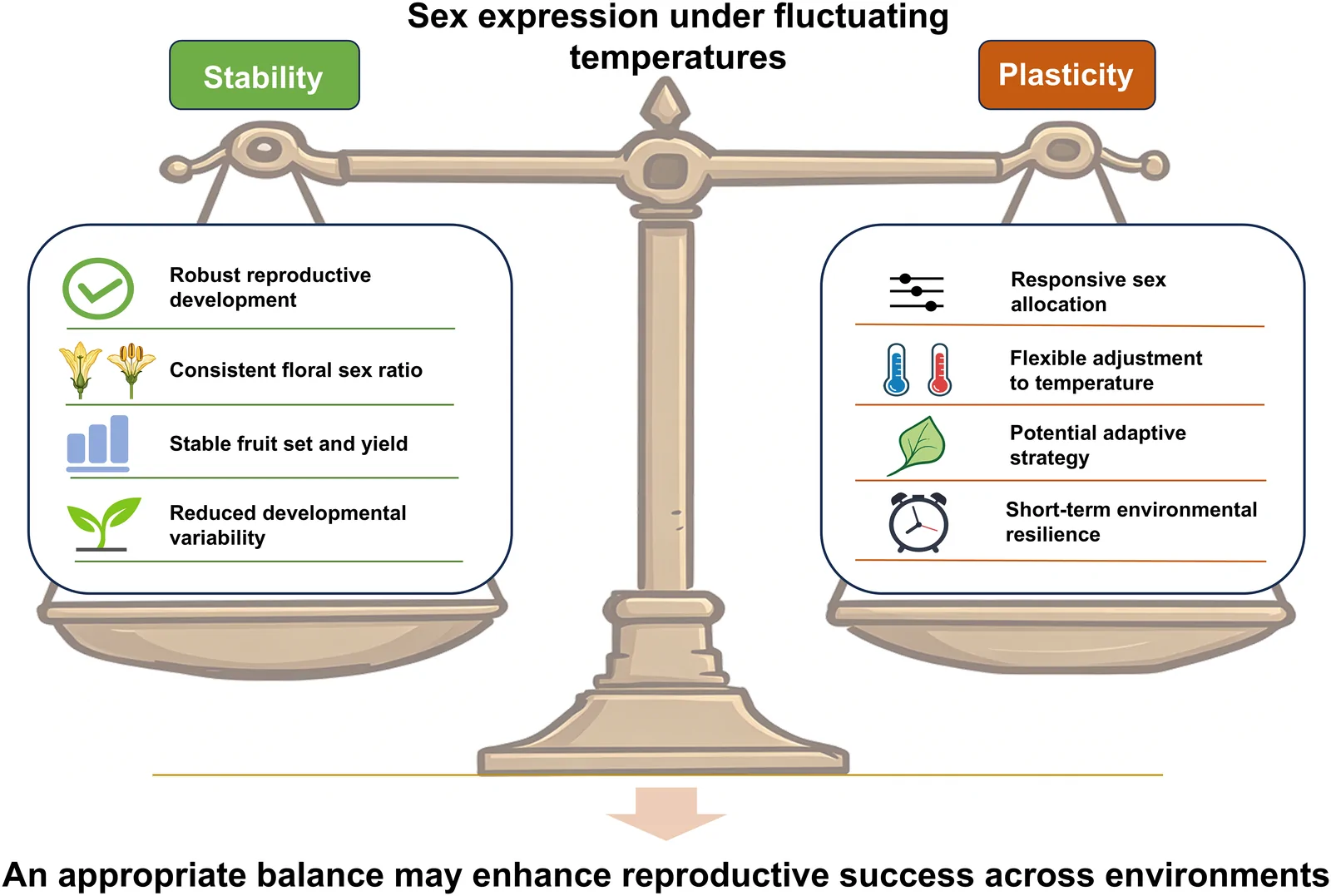
Stability–plasticity trade-off in plant sex expression under fluctuating temperatures. Stable sex expression supports predictable reproductive output, whereas plasticity allows adjustment of male and female allocation. Excessive plasticity may nevertheless cause sex-ratio imbalance or reduced reproductive performance. Adaptive significance therefore requires evidence from fitness, reproductive performance, genotype-by-environment variation, or evolutionary signatures rather than phenotypic shifts alone.

Stability and plasticity represent complementary but potentially conflicting properties of plant sex expression. Stable sex expression supports consistent mating structures and reproductive output, whereas plasticity allows adjustment of male and female functions in response to temperature, resource availability, and stress. Insufficient plasticity may constrain environmental responsiveness, whereas excessive plasticity may cause sex-ratio imbalance, mismatches with flowering or pollination, and unstable reproductive output (**Figure 3**). Their relative value therefore depends on ecological context, life-history strategy, and reproductive system (Brunet and Charlesworth, 1995; Morgan, 1992).

This trade-off is particularly relevant to crop production. In crops, plasticity can facilitate hormonal or temperature-based control of flower ratios, but excessive heat sensitivity may reduce female flowering, fruit set, and yield (Suijkerbuijk et al., 2025). Demonstrating adaptive value requires improved reproductive performance under the inducing environment, genotype-by-environment variation, or signatures of selection; such evidence remains limited.

### 4.3 Shared regulatory modules and lineage-specific wiring

Temperature-dependent sex expression may emerge when conserved environmental-response pathways are connected to lineage-specific sex-regulatory systems. Cucurbits, spinach, and *Cannabis* support a “shared signaling toolkit–lineage-specific wiring” model, in which ethylene- or GA-associated pathways feed into distinct downstream architectures (Li et al., 2019; Monthony et al., 2026; Zhang et al., 2021). These comparisons do not, however, demonstrate that cucurbit temperature-response mechanisms are conserved in other lineages.

The diversity of sex-determination systems in dioecious species such as asparagus and kiwifruit further illustrates that male-promoting and female-suppressing functions can be genetically organized in different ways (Harkess et al., 2020; Leite Montalvão et al., 2020). Cross-species comparisons should therefore focus not only on shared genes, but also on how temperature-responsive signals are connected to distinct sex-regulatory networks. Distinguishing genuinely shared modules from lineage-specific innovations may help identify the evolutionary origins of sexual plasticity and candidate alleles for temperature-stable sex expression.

## 5 Breeding and agricultural implications

### 5.1 Floral sex ratio management in cucurbit and horticultural crops

In cucurbits, female flower production and yield can be sensitive to temperature before floral identity is fixed. Temperature management should therefore target genotype-specific early developmental stages rather than post-anthesis periods (Zhao et al., 2026). However, effective temperature regimes are unlikely to be universally transferable. The optimal temperature range, treatment duration, and timing may vary among crops, genotypes, and cultivation systems. Management recommendations should therefore be based on genotype-specific developmental responses rather than on a single temperature threshold.

Chemical regulation can complement environmental management. Ethylene-releasing compounds such as ethephon can promote female flower formation, whereas GA treatments may increase male flower production or adjust floral sex ratios in seed-production materials (Li et al., 2021; Zhang et al., 2017). However, treatment outcomes depend strongly on genotype, concentration, developmental timing, and environmental conditions. Temperature and hormonal interventions should therefore be integrated as crop- and production-system-specific tools rather than applied as universally effective practices.

### 5.2 Breeding for temperature-stable sex expression

Breeding cultivars that maintain stable sex expression under heat represents an important translational goal, particularly for cucurbit crops grown during warm seasons or under protected cultivation. Desirable genotypes are not necessarily completely insensitive to environmental change, but should retain an appropriate proportion of female flowers, stable fruit set, and reliable yield under fluctuating or elevated temperatures.

Natural variation provides the foundation for breeding temperature-stable sex expression (Dhall et al., 2023; Lai et al., 2018; Prothro et al., 2013). Germplasm should be screened across temperature environments to identify stable phenotypes and associated alleles, while general heat-tolerance loci should not be assumed to regulate floral sex expression without direct phenotypic evidence.

Mechanistic studies have identified ACS/ACO, WIP, ERF-related factors, GA-responsive regulators, and epigenetically controlled loci as candidate components of floral sex regulation (Martin et al., 2009; Rodriguez-Granados et al., 2022). Nevertheless, few of these components have been shown to confer stable sex expression across realistic heat regimes. They should therefore be regarded as mechanistically informed candidate targets rather than validated targets for heat-resilient breeding.

A rigorous breeding pipeline should integrate phenotypic screening, genetic mapping, functional validation, and multi-environment evaluation. Practical targets should demonstrate causal control of floral sex fate, direct involvement in temperature responsiveness, robustness across genetic backgrounds and production environments, and no adverse effects on fertility or agronomic performance. Gene editing may facilitate causal testing, but breeding value must ultimately be established under greenhouse and field conditions.

### 5.3 Applications in hybrid seed production and controlled-environment agriculture

Temperature-dependent sex expression also has potential applications in hybrid seed production and controlled-environment agriculture, although floral sex regulation should be distinguished from broader fertility regulation. In monoecious cucurbits, floral sex ratios and flowering node positions affect parental-line arrangement, pollination efficiency, and seed-production management. In dioecious crops such as *Cannabis*, asparagus, and kiwifruit, early sex identification and maintenance of the desired sexual phenotype can likewise improve production efficiency (Chlosta et al., 2021; Harkess et al., 2020; Petit et al., 2020).

In hybrid seed-production systems, coordinated use of temperature and hormonal treatments may help adjust the abundance and timing of specific flower types. However, evidence from thermosensitive male sterility, flowering synchrony, and fertility stability in rice, wheat, and maize should be treated as conceptually related but distinct, because these systems concern fertility regulation rather than changes in floral sexual identity (Li et al., 2026; Schmidt et al., 2025, Yan et al., 2016).

Controlled environments enable precise testing of day/night temperatures, short-term heat events, developmental timing, and hormonal treatments. Combining environmental sensors with genotype-specific response models could support mechanism-guided floral sex management, provided that the resulting strategies are validated under commercial production conditions (Yuan et al., 2025).

## 6 Future perspectives

A major limitation of the current field is the fragmentation of evidence across biological levels. Most studies connect temperature exposure with only one or two of the following outcomes: hormonal or epigenetic responses, floral sex fate, whole-plant sex ratios, and reproductive performance. Future studies should integrate defined temperature regimes and developmental windows with cell-specific regulatory events, floral identity, and ultimately reproductive fitness or yield within the same experimental system.

### 6.1 From correlation to causality

Future studies should determine how temperature is perceived in responsive floral cell types, how these signals enter ethylene-, GA-, and epigenetic pathways, and which downstream genes causally determine male or female development rather than merely responding to temperature (Devani et al., 2019; Ding et al., 2020; Saini et al., 2022; Yang et al., 2015). Multi-omics and hormonal analyses can identify candidate pathways, whereas gene editing, VIGS, overexpression, mutant analysis, promoter assays, and hormone-complementation experiments will be required to establish causal links among temperature cues, molecular regulation, and floral sex fate.

### 6.2 Single-cell, spatial, and temporal resolution of floral sex fate

Temperature-dependent sex expression occurs within specific developmental windows and cell populations, whereas bulk transcriptomic analyses combine signals from shoot apical meristems, floral primordia, and differentiating floral organs. Single-cell and spatial transcriptomics could therefore identify temperature-responsive cell types and reveal how male- and female-biased developmental trajectories emerge (Denyer et al., 2019; Long et al., 2026; Sun et al., 2024). These approaches have already revealed cellular heterogeneity and spatial gene-expression patterns in plant development and environmental responses (Sang and Kong, 2024). In cucumber and melon, sampling shoot apices and early floral primordia under contrasting temperature regimes may identify temperature-responsive cell states and early sex-fate regulators. Spatial analyses could further determine whether ethylene biosynthesis genes, WIP-type genes, GA-signaling components, and epigenetic regulators change in specific regions of floral primordia. Integration with hormone quantification, epigenomic profiling, and functional validation will be essential to establish whether these molecular responses precede visible sexual differentiation.

Another unresolved question is whether transient temperature exposure creates a persistent developmental or epigenetic memory of sex expression (Lämke and Bäurle, 2017; Staacke et al., 2025). Changes in later-formed flowers may reflect irreversible determination of floral primordia already present during treatment rather than a true memory state. Demonstrating sex-expression memory will therefore require distinguishing developmental carryover from persistent molecular reprogramming and testing whether the effect extends to newly initiated meristems or subsequent generations.

### 6.3 Natural variation, QTL/GWAS, and elite alleles

Although temperature-responsive sex expression varies widely among genotypes, trait-specific QTL, GWAS, and population-genetic studies remain scarce (Faux et al., 2016; Petit et al., 2020; Prothro et al., 2013). Expanding these analyses across diverse germplasm will be essential for identifying alleles that specifically regulate sex-expression stability rather than general heat tolerance.

Genetic dissection and cross-study comparison are also constrained by inconsistent phenotyping. Female-node frequency, floral sex ratio, bisexual flower formation, bud abortion, and the number of flowers reaching anthesis represent distinct developmental outcomes and should not be used interchangeably. Standardized phenotyping should document developmental stage, node position, total and aborted flower buds, flowers reaching anthesis, and subsequent reproductive success under defined temperature regimes. In cucurbits, female-node ratio, first female-flower node, male and bisexual flower proportions, fruit set, and yield should be evaluated across temperature gradients. In dioecious plants such as *Cannabis*, spinach, and asparagus, relevant traits include sex stability, sex-reversal frequency, and floral organ development (Harkess et al., 2020).

QTL and GWAS results should subsequently be integrated with transcriptomic, methylomic, hormonal, eQTL, and mQTL data to refine candidate loci and identify their regulatory functions (Wang et al., 2020). Candidate alleles will nevertheless require functional validation and multi-environment breeding evaluation. Only loci that produce stable and agronomically beneficial effects across genetic backgrounds and temperature regimes are likely to have practical value.

### 6.4 Field-relevant temperature regimes and translational pipelines

**Figure 4:**
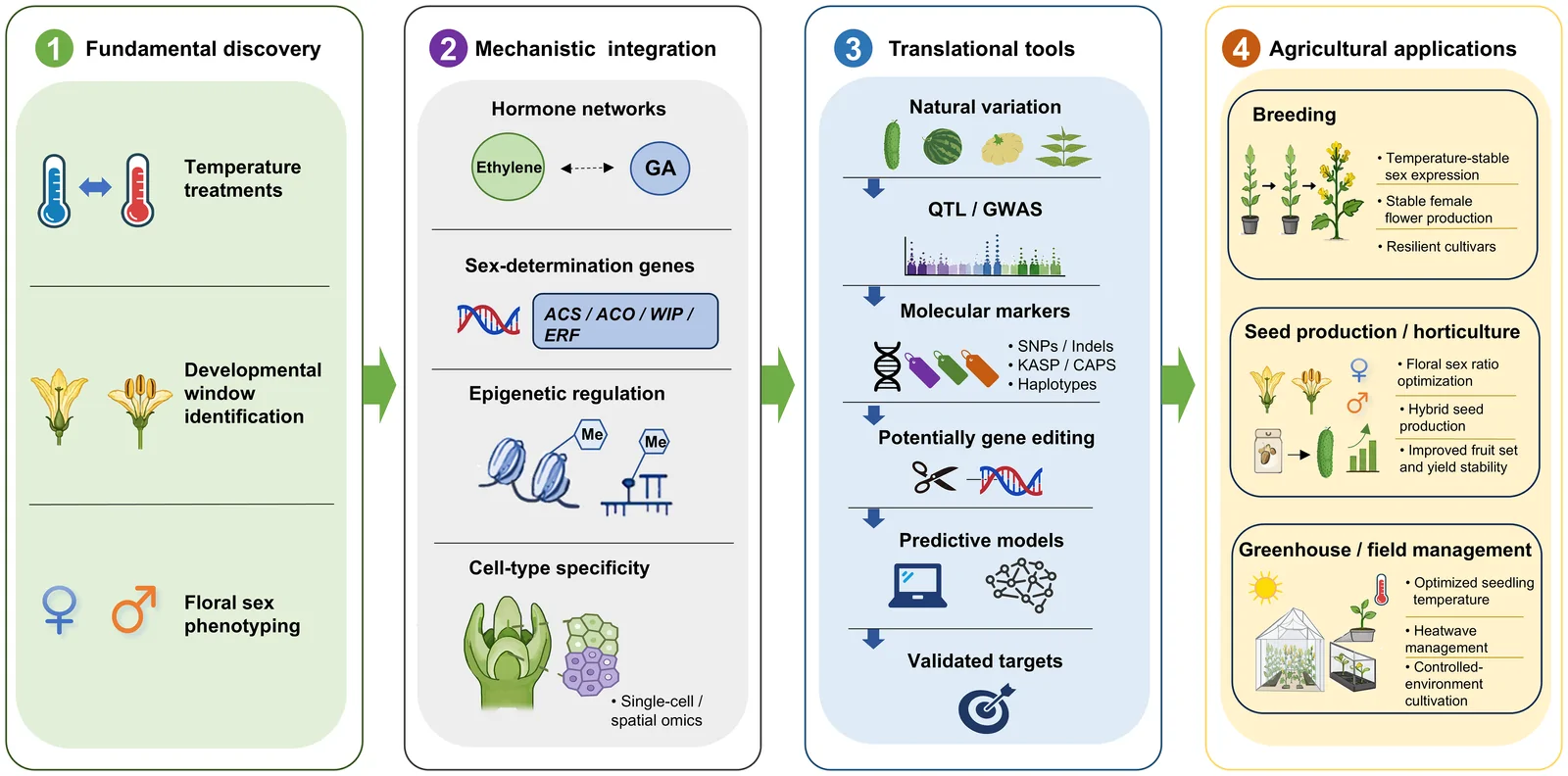
Translational roadmap from mechanistic understanding to breeding and cultivation management. Research should progress from the definition of temperature regimes, responsive developmental windows, and floral phenotypes to mechanistic validation, natural-variation analysis, and multi-environment evaluation. Candidate genes, alleles, and management interventions require causal validation across genetic backgrounds and realistic production environments before application in temperature-stable breeding, hybrid seed production, or cultivation management. Validated findings may support breeding for temperature-stable sex expression, floral sex-ratio management in hybrid seed and horticultural production, and greenhouse or field management under fluctuating temperatures. Some graphical elements were hand-drawn by the authors; others were created with the assistance of BioGDP.com (Jiang et al., 2025) and are used with permission.

Most studies rely on constant high- or low-temperature treatments, whereas field and protected-cultivation environments involve heatwaves, nocturnal warming, fluctuating day–night temperatures, and interactions between heat and other stresses (Jagadish et al., 2021; Staacke et al., 2025). Temperature-dependent sex expression should therefore be analyzed in relation to thermal intensity, duration, developmental timing, and genotype rather than as a simple binary contrast between high and low temperature. Future experiments should use field- or greenhouse-informed temperature profiles and incorporate humidity, water availability, multiple genotypes, and repeated production seasons (Praat et al., 2024). Evaluation should extend beyond floral sex ratios to flowering time, fertility, fruit set, seed production, and yield stability. Candidate genes and management interventions identified under controlled conditions should be advanced only when their effects remain stable under realistic production environments without compromising fertility or yield (**Figure 4**).

## 7 Conclusion

Temperature-dependent sex expression is a distinct form of reproductive plasticity in which temperature alters floral sex fate or sex-expression patterns before reproductive organ identity is fixed. Cucurbits provide the strongest evidence, with well-characterized ethylene-centered networks and emerging roles for GA and epigenetic regulation, although the causal path from temperature perception to sex fate remains incomplete. Spinach and *Cannabis* demonstrate hormone-responsive sexual plasticity, whereas most other non-model systems provide indirect evidence or describe sex-specific stress responses. Progress will require causal, cell-resolved analyses, standardized phenotyping, and field-relevant temperature experiments across diverse germplasm. Such advances could support temperature-stable sex expression, hybrid seed production, and precise reproductive management in cucurbit and other horticultural crops.
